# The Use of Lactic Acid Bacteria as a Probiotic in Swine Diets

**DOI:** 10.3390/pathogens4010034

**Published:** 2015-01-27

**Authors:** Fengjuan Yang, Chengli Hou, Xiangfang Zeng, Shiyan Qiao

**Affiliations:** State Key Laboratory of Animal Nutrition, China Agricultural University, No. 2, Yuanmingyuan West Road, Beijing 100193, China; E-Mails: yangfj0115@gmail.com (F.Y.); houchengli@163.com (C.H.); ziyangzxf@163.com (X.Z.)

**Keywords:** probiotics, lactic acid bacteria, pigs, antibiotic alternatives

## Abstract

As the resistance of pathogens to antibiotics and the possibility of antibiotic residues in animal products attract increasing attention, the interest in the use of alternatives to in-feed antibiotics has been growing. Recent research with Lactic acid bacteria (LAB) in pigs suggests that LAB provide a potential alternative to antibiotic strategies. LAB include *Lactobacillus* species, *Bifidobacterium* spp, *Bacillus* spp, and some other microbes. LAB can adjust the intestinal environment, inhibit or kill pathogens in the gastrointestinal tract and improve the microbial balance in the intestine, as well as regulate intestinal mucosal immunity and maintain intestinal barrier function, thereby benefiting the health of pigs. The related mechanisms for these effects of LAB may include producing microbicidal substances with effects against gastrointestinal pathogens and other harmful microbes, competing with pathogens for binding sites on the intestinal epithelial cell surface and mucin as well as stimulating the immune system. In this review, the characteristics of LAB and their probiotic effects in newborn piglets, weaned piglets, growing pigs and sows are documented.

## 1. Introduction

Antibiotics have been widely used for growth promotion and prevention of diarrhea in farm animals [1]. As a common additive used in livestock feeds, antibiotics contribute to an improved economic efficiency. However, the negative effects of antibiotics have become increasingly prominent. Consumers are increasingly concerned about antibiotic residues in meat products [2]. Furthermore, it has been suggested that the continuous use of antibiotics may increase bacterial resistance, which can threaten the health of both animals and humans [3]. Therefore, the use of antibiotics as growth promoters has been banned in many countries, such as the European Union and Japan. In addition, other countries including China and the United States are banning or planning to ban the inclusion of antibiotics in swine diets.

Lactic acid bacteria (LAB) have been suggested to be an alternative strategy to antibiotic growth promoters [4]. LAB comprise a group of gram-positive, acid-tolerant, generally non-sporulating, non-respiring rod shaped (bacillus), or spherical (coccus) bacteria that are associated by their common metabolic and physiological characteristics. These bacteria produce lactic acid as the major metabolic end-product of carbohydrate fermentation. This trait has linked LAB with food fermentation, as acidification inhibits the growth of spoilage agents. Furthermore, lactic acid and other metabolic products contribute to the organoleptic and textural profile of a food item [5]. LAB includes various major genera, including *Lactobacillus* spp, *Bifidobacterium* spp, *Lactococcus* spp, *Lactosphaera* spp, *Leuconostoc* spp, *Melissococcus* spp, *Oenococcus* spp, *Pediococcus* spp, *Streptococcus* spp, and *Enterococcus* spp [6,7].

In recent years, multiple reports have described the beneficial effects of LAB, such as regulation of the intestinal microflora, inhibition or prevention of pathogens in the gastrointestinal tract (GIT), enhancement of intestinal mucosal immunity and maintaining intestinal barrier function [8,9,10,11,12]. The aim of this review is to systematically review and update the evidence on the efficacy of using LAB in pig diets.

## 2. Applications of Lactic Acid Bacteria in Pigs

Studies in LAB applied to replace antibiotics in pigs have noted LAB have a lot of beneficial effects. We have constructed a summary table (Table 1) from some literatures, in order to provide a visualized overview of the reported LAB trains used in pigs.

### 2.1. Applications of LAB in Neonatal Piglets

The neonatal period is a critical time in piglet ontogeny, due to the fact that the GIT and immune system have yet to fully develop [13]. These deficiencies result in low disease resistance in piglets and make them vulnerable to invasion by pathogenic microorganisms. A serious infection or stress reaction in the neonate has negative impact on piglets, thus affecting the whole process of individual development [14].

Supplementation of LAB in neonatal piglets can regulate the formation of the piglet gut microflora, thus benefiting the health of piglets [13,14]. Liu *et al.* [15] found that oral administration of *L. fermentum* I5007 in formula-fed piglets (dosed daily with 6 × 10^9^ CFU/mL of *L. fermentum* I5007 dissolved in 3 mL of 0.1% peptone water once a day for 14 days *vs.* just 0.1% peptone water in control group) favored intestinal development and reduced the number of potentially enteropathogenic *Escherichia* spp and *Clostridium* spp in neonatal piglets. This was consistent with an earlier study showing that piglets provided with LAB (enteral feeding for 2 days with formula with LAB *vs.* porcine colostrum or formula groups) had a lower density of the potential pathogen *Clostridium perfringens* [14]. In addition, commensal *Lactobacillus* bacteria were more closely associated with enterocytes along the villus-crypt in piglets treated with LAB [14].

LAB have been shown to enhance intestinal barrier function [15,16]. A dysfunction in the intestinal barrier plays a major role in the pathophysiology of a variety of gastrointestinal disorders. Previous research demonstrated that various strains of *Lactobacillus* were responsible for different modulations of cell layer integrity and could attenuate the barrier disruption (rearrangement of ZO-1) caused by *Salmonella* LPS administration [17]. Another study indicated that oral administration of *L. fermentum* I5007 decreased the mRNA expression of the inflammatory cytokine IL-1β and increased the concentrations of butyrate [15], which help strengthen the intestinal barrier and defend against pathogenic microbes [18]. Wang *et al.* [19] also studied this strain and revealed that *L. fermentum* I5007 improved weight gain and feed conversion, decreased the occurrence of diarrhea, enhanced T-cell differentiation and induced cytokine expression in the ileum of piglets.

### 2.2. Applications of LAB in Weaned Piglets

During weaning, piglets are faced with a considerable amount of psychological stress induced by changes in feed and the environment. LAB can relieve weaning stress, prevent diarrhea and promote growth of piglets during and after weaning [10,20,21]. In addition to improving the intestinal microbiota of piglets, inclusion of *Enterococcus faecium* significantly improved growth and feed conversion of weaning pigs [22,23]. LAB complexes, such as a combination of *E. faecium*, *L. acidophilus*, *Pediococcus pentosaceus* and *L plantarum* (a basal diet without antibiotics or probiotics was used as control, and the other 3 groups were fed the control diet supplemented with 600 ppm of one of three different LAB complexes) increased feed intake and weight gain and improved feed conversion [24]. Yu *et al.* [25] demonstrated that *L. fermentum* I5007 (a basal diet with *L. fermentum* supplementation as the experimental group *vs.* a basal diet without antibiotics or *L. fermentum* as the control group) colonized and adhered to the GIT epithelium forming a protective membrane against pathogenic microbes while at the same time modulating immunity along with promoting the expression of MUC2 and MUC3. In addition, *L. fermentum* I5007 exhibited additional effects in alleviating weaning stress syndrome by enhancing the levels of proteins involved in energy metabolism, lipid metabolism, cell structure and mobility, protein synthesis, immune response [26], and improved the anti-oxidative defence system [27], thereby facilitating cellular proliferation and depressing apoptosis.

### 2.3. Applications of LAB in Growing-Finishing Pigs

As growing-finishing pigs have a mature GIT, with high digestive enzyme activity, immune capacity and disease resistance, the influence of LAB in growing-finishing pigs is relatively limited. Supplementation of a LAB mixture (based on *Bacillus lichenformis* and *B. subtilis*, probiotic application group fed a basal diet with LAB mixture *vs.* control group fed a basal diet with antibiotic used as) improved weight gain and reduced mortality of growing-finishing pigs [28]. Ohashi *et al.* [29] evaluated the effect of feeding yoghurt, prepared with *L. bulgaricus* strain 2038 (three female pigs fistulated at the cecum were fed 250 g of this yoghurt for 2 weeks; the whole experiment was divided to pre-administration period, administration period and post-administration period), on indigenous lactobacilli in the pig cecum and found that continuous consumption of this strain will stimulate the growth of some indigenous lactobacilli and alter the composition of the lactobacilli. *L. plantarum* ZJ316 (the control group was fed a diet supplemented with the antibiotic mequindox, three groups with different *L. plantarum* levels and a group with a mixture of mequindox and *L. plantarum*) was also found to improve pig growth and pork quality. The probiotic mechanism was related to the inhibition of the growth of opportunistic pathogens and promotion of increased villus height [30].

### 2.4. Applications of LAB in Sows

Although there are relatively few studies about the application of LAB in sows, it is very important to conduct research in this field. In a recent study, the effects of *L. johnsonii* XS4 (control group received basal diet and experiment group received the same diet supplemented with *L. johnsonii* XS4, from 90th day of pregnancy to the weaning day at 25th day of lactation) on reproductive performance, gut environment, and blood biochemical and immunological indexes of sows were investigated. The results showed that administration of *L. johnsonii* XS4 in diets towards the end of pregnancy and during lactation had positive effects on the performance of sows, increasing litter weight at birth, 20-day litter weight, the number of piglets at weaning and weaning litter weight, along with a significant increase in serum IgG levels and a decrease in alanine aminotransferase concentrations [31]. Lactina, a mixture of *Streptococcus thermophiles*, *E. faecium*, *L. bulgaricus*, *L. acidophilus*, *L. helveticus* and *L. plantarum*, supplemented both to sow and piglet diets, increased complement activity in piglets at 5 days of age compared with a control group, while the addition of Lactina to sows only or to piglets only did not produce any significant effects [32]. Another probiotic mixture of *B. licheniformis* and *B. subtilis* (normal feed plus the probiotic mixture *vs.* untreated control group) was shown to improve sow feed intake and decrease sow weight loss during the sucking period [8].

### 2.5. Supplementation Stage and Optimum Dose of LAB in Pigs

Many studies have been conducted on optimal supplementation strategies in pigs. The effects of *L. plantarum* ZJ316 on pig growth at a dose of 1 × 10^9^ CFU/day were more pronounced than a dose of 5 × 10^9^ CFU/day or 1 × 10^10^ CFU/day [30]. Zhu *et al.* [33] reported the effects of *L. rhamnosus* ATCC7469 on serum IL-17 production and intestinal T-cell responses in pigs challenged with *E. coli* were dose-dependent, showing that serum concentrations of IL-17 and the percentage of ileal intraepithelial CD3^+^CD4^−^CD8^+^ cells increased in the high-dose (1 × 10^11^ CFU/mL) piglets, but not the low-dose (1 × 10^9^ CFU/mL) piglets [33]. Furthermore, Yu *et al.* [25] fed weaned piglets with diets containing 3.2 × 10^6^ CFU/g, 5.8 × 10^7^ CFU/g or 2.9 × 10^8^ CFU/g of *L. fermentum* I5007. Their results showed that a dose of 5.8 × 10^7^ CFU/g maximized the digestibility of crude protein among the different concentrations of *L. fermentum*.

The supplementation stages of *L. reuteri* I5007 has also been studied by oral administration (1.7 × 10^10^ CFU/day for each piglet) either daily for 4 days starting on day 1 or every 4th day from day 1 to 17. The data showed that piglets in the prolonged duration of treatment (every 4th-day group) had the highest abundance of mRNA for TGF-β and the lowest for IFN-γ [34].

**Table 1:** Application and probiotic effects of lactic acid bacteria in swine.

Application	Strain	Probiotic Effects	References
Neonatal piglets	*L. fermentum* I5007	increase average dairy gain, improve intestinal immunty	[15]
	*E. faecium* EF1	induce a strong anti-inflammatory response in the small intestine	[35]
	*L. casei*	decrease the number of *E. coli* colonising jejunal mucosa of gnotobiotic piglets	[36]
Weaned piglets	*L. reuteri* BSA131	improve weight gain and feed conversion, reduce the number of fecal coliform	[37]
	LAB complexes	improve growth performance, increase apparent ileal digestibility of crude protein, crude fiber and organic matter	[24]
	*L.* rhamnosus GG	ameliorate diarrhea, increase sIgA concentrations and attenuate the elevation of serum IL-6 induced by *E. coli* K88	[38]
	*L. amylovorus* and *E. faecium*	increase monounsaturated and polyunsaturated fatty acids, modify and improve the fatty acid profile of pig meat	[39]
Growing-finishing pigs	*L. plantarum* ZJ316	improve weight gain and feed conversion, reduce the incidence of diarrhea, improve meat quality	[30]
	LAB complexes	increase average dairy gain, improve feed conversion, increase digestibility of crude protein and organic matter	[40]
	*E. faecium* SF68	increase nutrient digestibility and decrease faecal NH_3_-N, H_2_S and volatile fatty acid concentrations	[23]
Sows	*L. johnsonii* XS4	increase litter weight at birth, 20 d litter weight, the number of piglets at weaning and weaning litter weight, show an increase in serum IgG levels	[31]
	*E. faecium* SF68	increased intestinal IgA secretion both in sows and piglets	[41]

## 3. Properties or Action Modes of Lactic Acid Bacteria

### 3.1. Survival and Adhesion within the Gastrointestinal Tract

To behave as a probiotic, LAB must first be able to survival passage though the upper GIT, meaning that LAB must have the characteristics of resistance to increased acidity from inorganic acid production (e.g., hydrochloric acid) and pancreatic enzymes [42]. The most commonly used probiotics are strains of LAB such as *Lactobacillus*, and *Bifidobacterium*, which are known to withstand gastric acid, bile salts and pancreatic secretions, to adhere to colonic mucosa and readily colonize the intestinal tract [43]. For example, *Lactobacillus reuteri* I5007, initially known as *L. fermentum* I5007, was selected from over 7000 native Lactobacilli colonies according to criteria including tolerance to heat, low pH, and bile salts, as well as storage stability and antagonism to pathogenic agents [44]. Charteris *et al.* [36] found that *Lactobacillus* and *Bifidobacterium* showed a moderate tolerance to acid pH during 1.5 h of incubation which was decreased after 2 h [45]. Previous studies have pointed out that acid resistance appeared to be mediated by membrane ATPases as described for *L. acidophilus* [46] and bile resistance was mediated by bile salt hydrolysis in *L. reuteri* [47].

Secondly, LAB as probiotics should have the potential to adhere to intestinal epithelial cells [42]. Adhesion of a probiotic strain to the GIT is important for bacterial colonization, pathogen exclusion, and interaction with host cells for the protection of epithelial cells or immune modulation [48]. *L. reuteri* I5007 showed strong adhesion to porcine intestinal mucus and several cell lines such as Caco-2 cells, IPEC-J2 cells and IEC-6 cells [49,50]. Other LAB strains also have the capacity to adhere to mucus and the intestinal epithelial cells [50,51,52]. Mechanisms of adherence to an epithelial surface involve receptor-specific binding and charge as well as hydrophobic interaction. LAB commonly express cell surface hydrophobicity, contact angle and adhesion to xylene [53]. This may facilitate adhesion to mucus. Furthermore, Cell Surface Proteins have been shown to mediate adhesion to mucus by various LAB [54]. Interestingly, LAB showed no host specificity in adhesion to intestinal mucus, but differed between the different compartments of the GIT [55].

### 3.2. Antibacterial and Bactericidal Effects

One of the most important modes of action of LAB is antimicrobial activity through inhibition of the adhesion of pathogenic bacteria [42]. The lactic acid produced by LAB contributes to an acidic environment in the GIT which partly influences growth of pathogenic microorganisms. What’s more, LAB commonly produce bacteriocins which are peptides with bactericidal activity usually against strains of closely related species and can inhibit growth or adhesion of harmful bacteria. A protein secreted from *L. acidophilus* was reported to inhibit the gastric pathogen *Helicobacter pylori* and supplementation of *Saccharomyces boulardii* to rumen fluid eradicated *Escherichia coli* O157:H7 [46,56]. Li *et al.* [41] reported that *L. fermentum* I5007 had a strong competitiveness against both *E. coli* K88 and *Salmonella typhimurium* and could adhere to Caco-2 cells and porcine intestinal mucosa [50].

LAB can inhibit pathogenic bacteria by competing for nutrients in the gut or for binding sites on the intestinal epithelium [57]. As most intestinal pathogens must adhere to the intestinal epithelium to colonize in the intestine and produce diseases [58], some LAB strains have been chosen as probiotics specifically based on their ability to adhere to the intestinal epithelium and thus compete with pathogens for binding sites [59].

Another mechanism to inhibit pathogens in the gut is via increasing production of intestinal mucins which may protect the epithelial cells by functioning as a physicochemical barrier. *L. plantarum* 299v was shown to increase mRNA expression of MUC2 and MUC3 in HT29 intestinal cells, and this led to inhibition of adhesion of enteropathogenic *E. coli* [60].

### 3.3. Antioxidation and Immunomodulation

Some LAB strains produce antioxidants and influence the immue system. It is well known that oxidative damage forms part of the pathogenesis for many chronic diseases. *Bifidobacterium longum* ATCC 15708 and *L. acidophilus* ATCC 4356 inhibited linoleic acid peroxidation and scavenged free radicals. *L. fermentum* I5007 also demonstrated the ability to scavenge free radicals *in vitro* [61]. LAB provides defense by inducing anti-inflammatory cytokines and reducing proinflammatory cytokines from intestinal epithelial cells [62,63], but certain LAB will enhance the gut inflammatory immune response [64]. For instance, *L. lactis* and *L. bulgaricus* induced an increase in IgA^+^ cells entering the IgA cycle but not CD4^+^ cells. However, *L. casei* and *L. plantarum* were able to increase IgA^−^ cells and CD4^+^ cells [65]. In addition, *L. casei* Shirota induced production of the proinflammatory cytokine IL-12 with subsequent production of IFN-γ in murine splenocytes [66]. The properties of immunomodulation appear to be strain dependent.

## 4. Safety

The industrial importance of the LAB is evidenced by their “Generally Regarded as Safe (GRAS)” status. Studies of LAB both *in vitro* and *in vivo* indicate that they are safe for livestock and human consumption [67,68]. However, plasmids in some strains of LAB have been shown to encode for antibiotic resistance genes [49,69,70]. For instance, *L. reuteri* ATCC 55730, a commercially available probiotic strain, was demonstrated to carry potentially transferable resistance traits for tetracycline and lincomycin. However it has been replaced by *L. reuteri* DSM 17938, in which the two resistance plasmids have been removed without losing any probiotic characteristics [70]. At the same time, the taxonomy of several LAB has been reconstructed during the last decade, and the use of modern polyphasic taxonomy has reclassified several probiotic strains [6,49,71]. Generally, LAB strains carry a very low risk of causing infection. Many related products have been traditionally used over generations, and have been proven to be safe.

## 5. Conclusions

In conclusion, the available data from studies and applications of LAB in pigs clearly indicate that LAB have great potential as alternatives to in-feed antibiotics. However, LAB are not a single entity. Different LAB strains even of the same species may have different metabolic effects which in turn affect performance and the immune system of the host. Therefore, randomized, double-blind, placebo-controlled, case-controlled studies on the efficacy of LAB preparations, as well as optimal supplementation stages and doses, are needed.
